# Supplementary material for: Polycomb-associated and Trithorax-associated developmental conditions—phenotypic convergence and heterogeneity
Source: Eur J Hum Genet. 2025 Jan 22;33(11):1414–21. doi: 10.1038/s41431-025-01784-2 (PMC12583576; doi:10.1038/s41431-025-01784-2)
Supplement: Supplementary file 1 — Supplementary Figures [file 41431_2025_1784_MOESM1_ESM.docx]

**Supplementary Figure 1**

Gene list curation and cohort curation


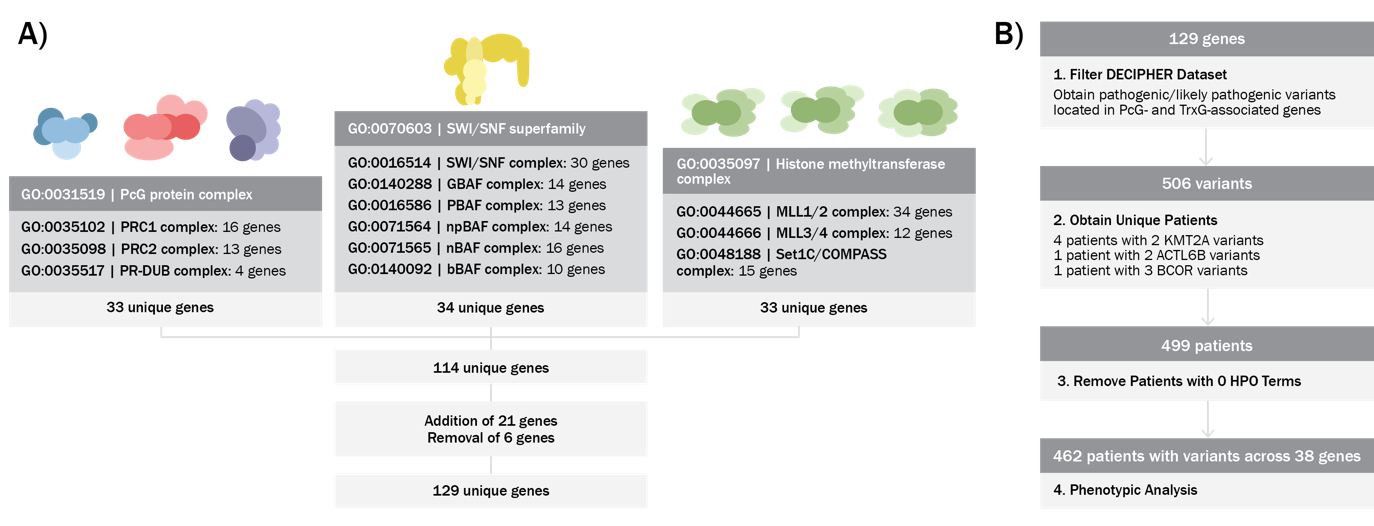


A) PcG / TrxG gene curation. Gene ontology terms relevant to PcG- and TrxG-mediated epigenomic regulation were selected to identify relevant complexes and genes.

B) DECIPHER dataset filtering process. The full DECIPHER dataset was filtered to identify individuals with a pathogenic or likely pathogenic variant located in a PcG- or TrxG-associated gene.

**Supplementary Figure 2**

Distributions of total HPO term numbers in PcG/TrxG and comparison groups


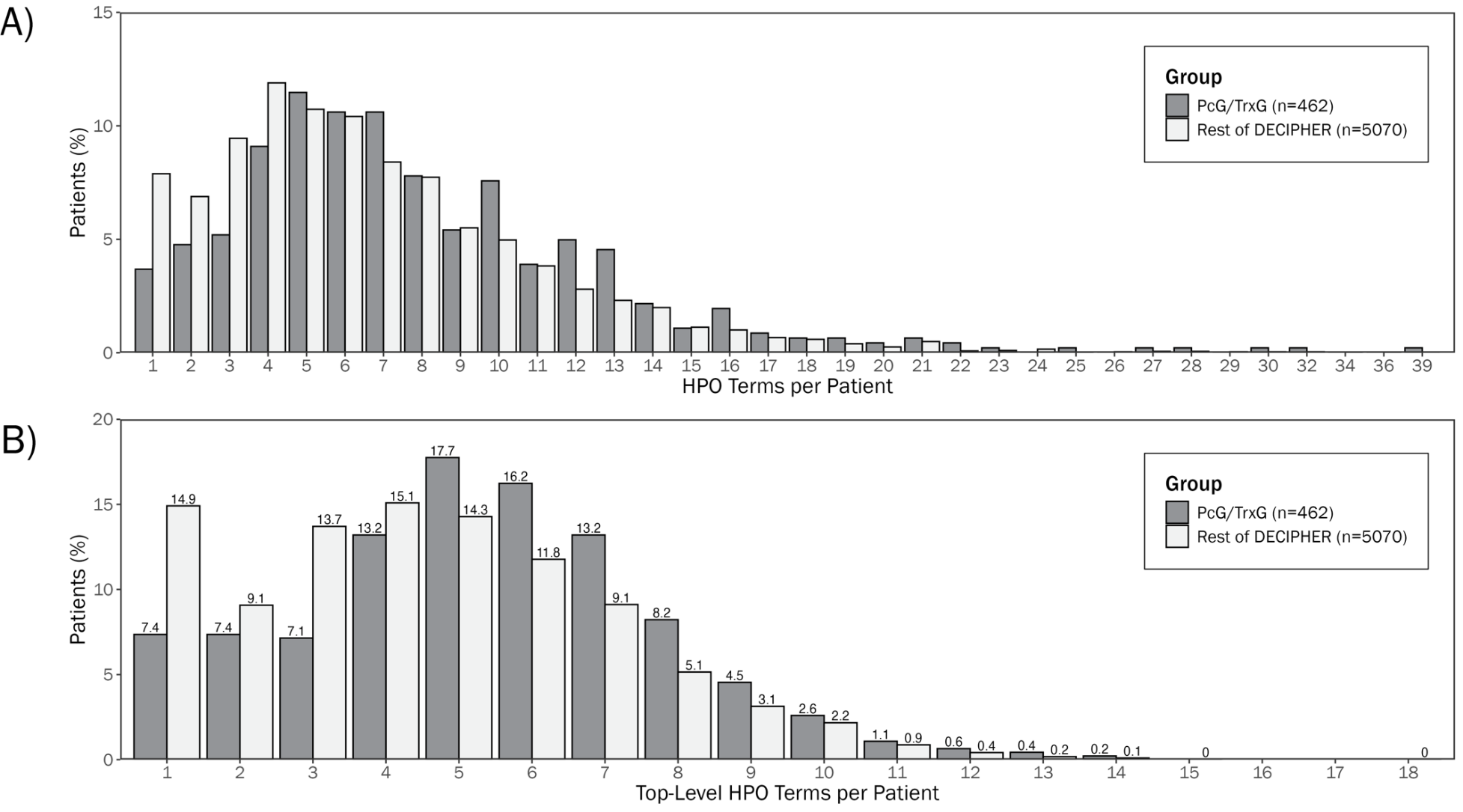


A) Number of raw HPO terms reported within DECIPHER per individual (case and comparison groups).

B) Number of top-level HPO terms per individual after propagation (case and comparison groups).

**Supplementary Figure 3**

Distribution of top-level HPO Terms in PcG and TrxG-associated conditions, by genetic diagnosis


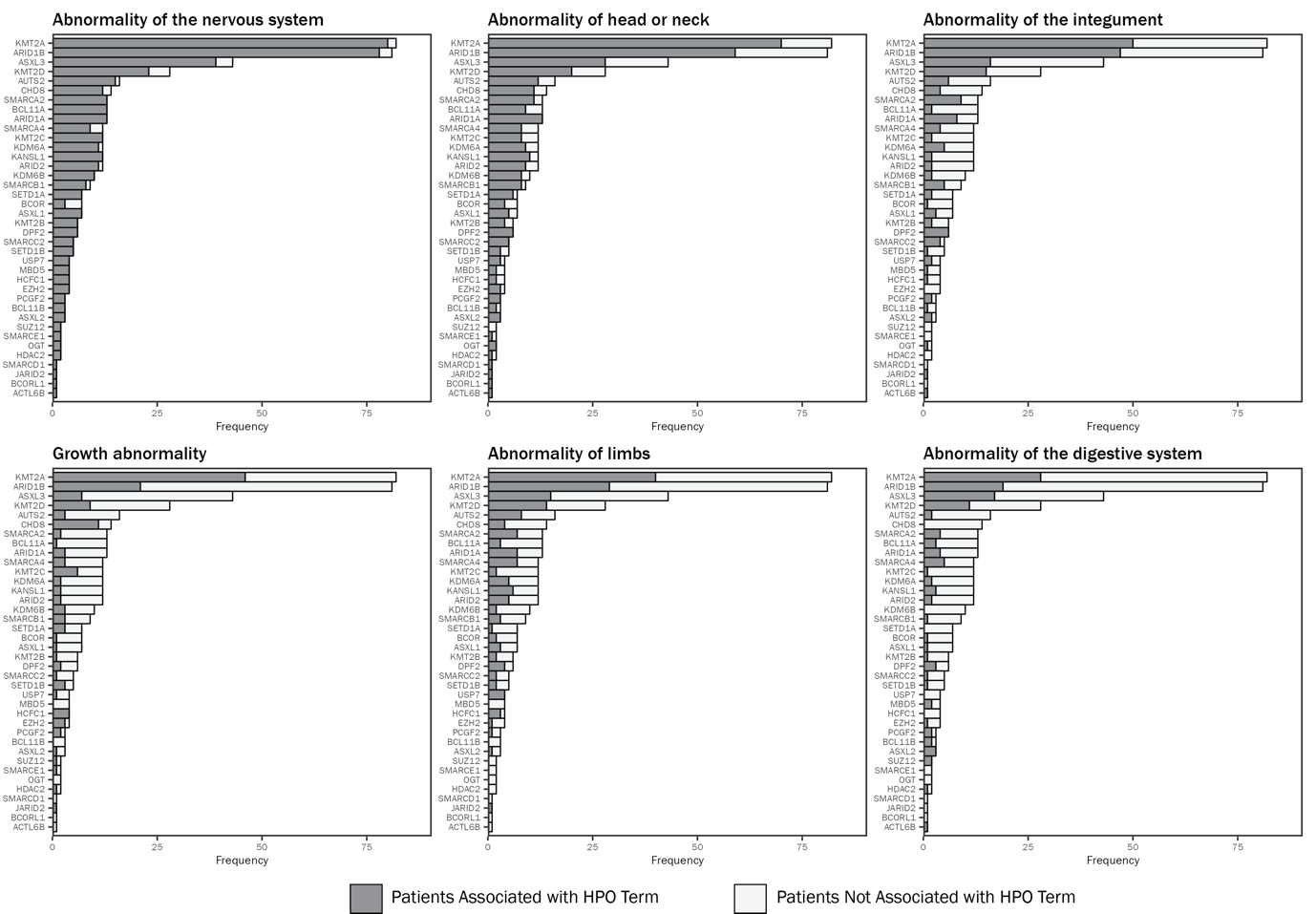


Each bar plot represents a top-level phenotype term that was identified as significantly enriched in the PcG/TrxG cohort.

For each top-level term, the number of individuals with and without this reported phenotype (after propagation) is displayed for each gene group within the PcG/TrxG cohort.

**Supplementary Figure 4**

HPO term list sizes for gene-level and patient-level clusters


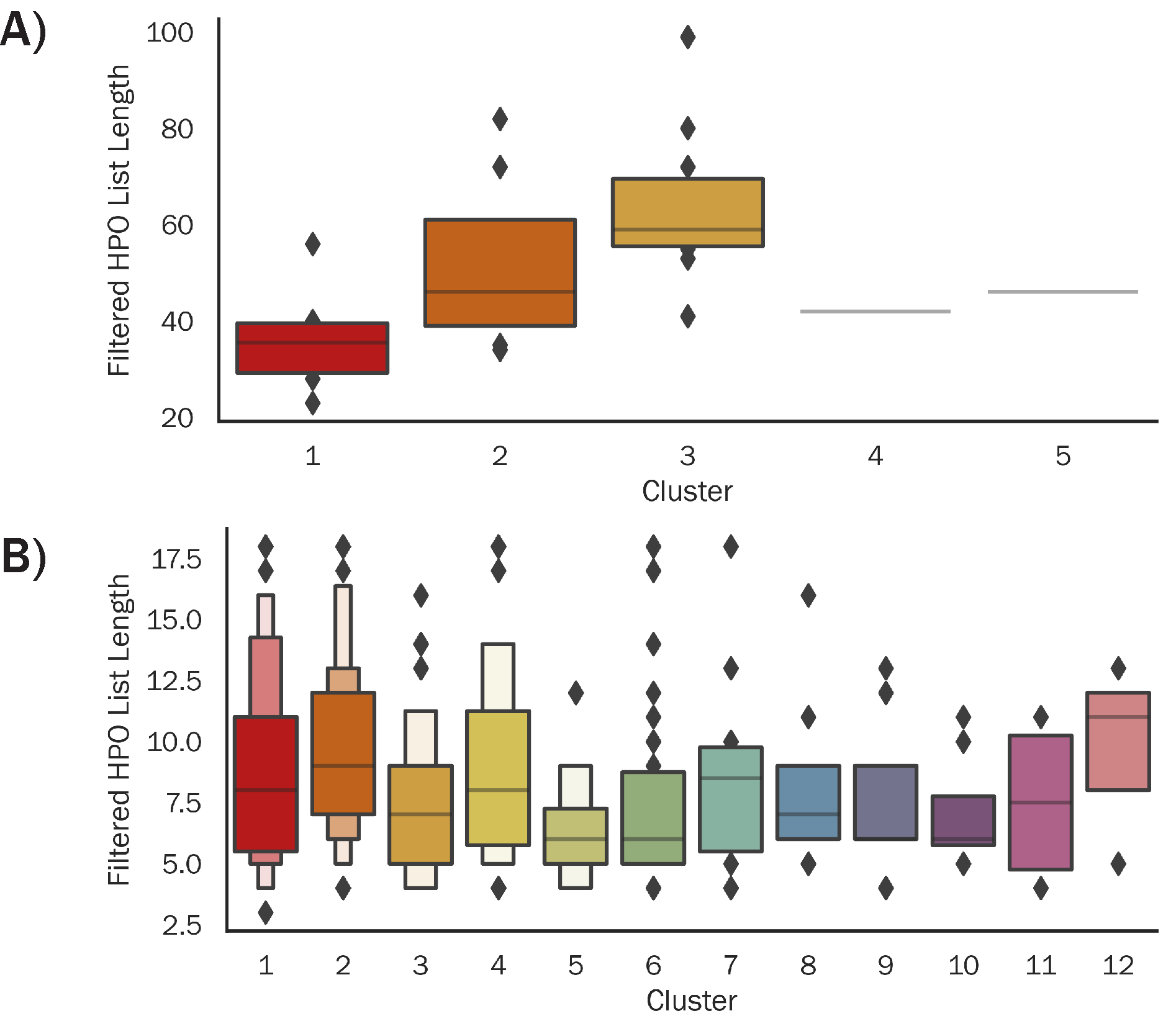


A) Boxen plot showing filtered HPO list length for each gene in each gene-level cluster. Centre line represents median value, with boxes either side successively capturing 50% of the remaining datapoints. Outliers are represented as diamonds.

B) Boxen plot showing filtered HPO list length for each individual in each patient-level cluster. Centre line represents median value, with boxes either side successively capturing 50% of the remaining datapoints. Outliers are represented as diamonds.

**Supplementary Figure 5**

Distribution of patient-level cluster memberships within each gene group.
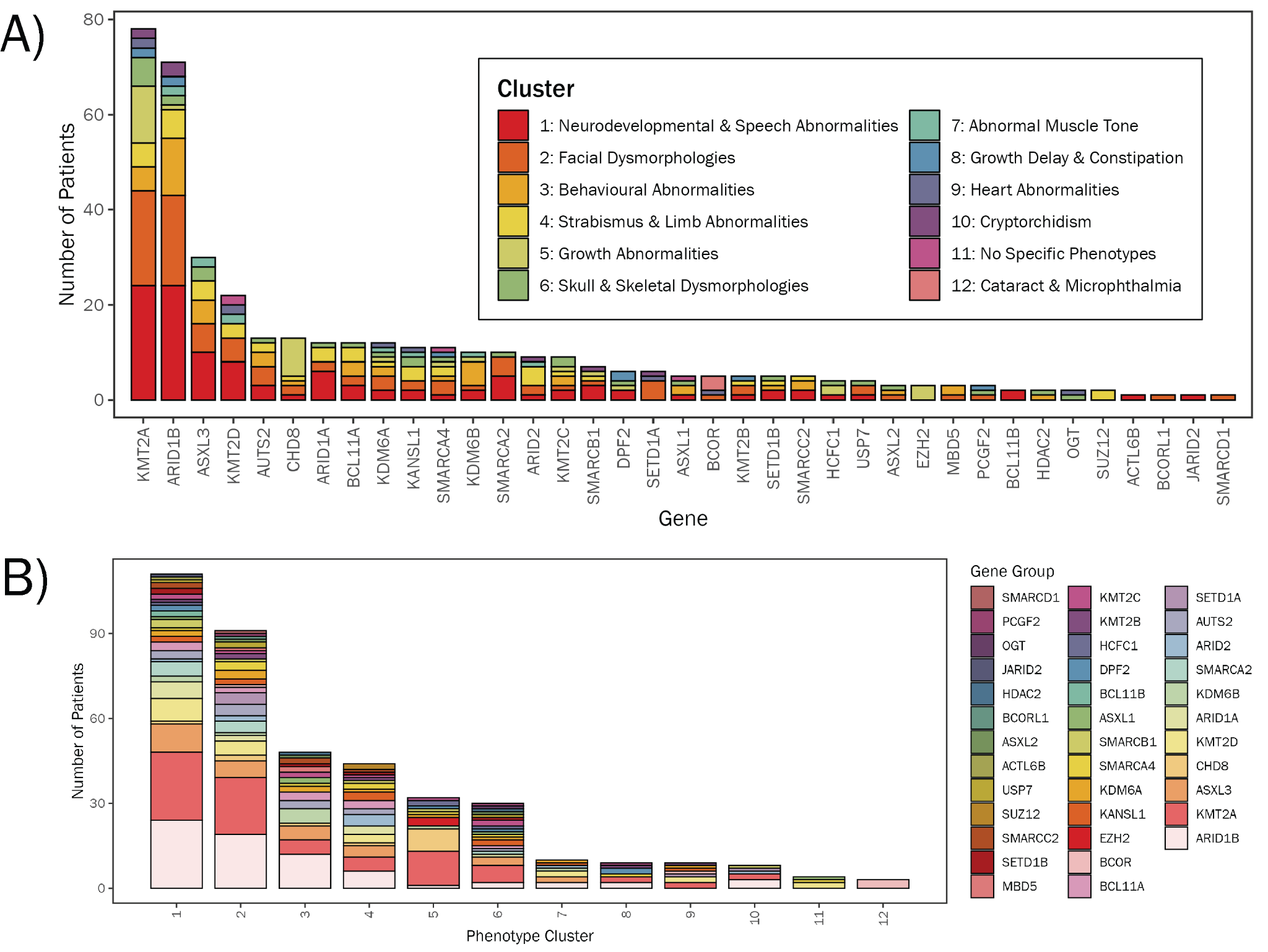


A) Stacked bar plot showing proportion of each gene group that belongs to each identified phenotypic cluster.

B) Stacked bar plot showing proportion of each identified phenotypic cluster that belongs to each gene group.
